# Electro-Acupuncture Attenuates Chronic Stress Responses *via* Up-Regulated Central NPY and GABA_*A*_ Receptors in Rats

**DOI:** 10.3389/fnins.2020.629003

**Published:** 2021-01-26

**Authors:** Yu Yang, Haijie Yu, Reji Babygirija, Bei Shi, Weinan Sun, Xiaojiao Zheng, Jun Zheng

**Affiliations:** ^1^Department of Physiology, School of Life Sciences, China Medical University, Shenyang, China; ^2^Department of Cardiology, First Affiliated Hospital, China Medical University, Shenyang, China; ^3^Department of Surgery, Medical College of Wisconsin and Zablocki VA Medical Center, Milwaukee, WI, United States

**Keywords:** stress, functional dyspepsia, electroacupuncture, neuropeptide Y, gamma-aminobutyric acid A receptor

## Abstract

Stress can increase the release of corticotropin-releasing factor (CRF) in the hypothalamus, resulting in attenuation of gastric motor functions. In contrast, central neuropeptide Y (NPY) can reduce the biological actions of CRF, and in turn weaken stress responses. Although electroacupuncture (EA) at stomach 36 (ST-36) has been shown to have anti-stress effects, its mechanism has not yet been investigated. The effect of EA at ST-36 on the hypothalamus-pituitary-adrenal (HPA) axis and gastrointestinal motility in chronic complicated stress (CCS) conditions have not been studied and the inhibitory mechanism of NPY on CRF through the gamma-aminobutyric acid (GABA)_*A*_ receptor need to be further investigated. A CCS rat model was set up, EA at ST-36 was applied to the bilateral hind limbs every day prior to the stress loading. Further, a GABA_*A*_ receptor antagonist was intracerebroventricularly (ICV) injected daily. Central CRF and NPY expression levels were studied, serum corticosterone and NPY concentrations were analyzed, and gastric motor functions were assessed. CCS rats showed significantly elevated CRF expression and corticosterone levels, which resulted in inhibited gastric motor functions. EA at ST-36 significantly increased central *NPY* mRNA expression and reduced central *CRF* mRNA expression as well as the plasma corticosterone level, helping to restore gastric motor function. However, ICV administration of the GABA_*A*_ receptor antagonist significantly abolished these effects. EA at ST-36 upregulates the hypothalamic NPY system. NPY may, through the GABA_*A*_ receptor, significantly antagonize the overexpressed central CRF and attenuate the HPA axis activities in CCS conditions, exerting influences and helping to restore gastric motor function.

## Introduction

The pathogeneses of functional gastrointestinal disorders (FGIDs), such as functional dyspepsia (FD), are highly related to stress in humans (Levy et al., 2006; Talley et al., 2015). Gastrointestinal (GI) dysmotility might develop as a result of the accumulation of repeated or continuous stress in some individuals.

Corticotropin-releasing factor (CRF) in the central nervous system plays a significant role in mediation of stress-induced GI dysmotility (Lenz et al., 1988; Tache and Bonaz, 2007). Acute restraint stress (ARS) inhibits gastric motility *via* sympathetic pathways and central CRF type 2 (CRF_2_) receptors (Nakade et al., 2005). In the chronic complicated stress (CCS) rat model, delayed gastric emptying (GE) and attenuated gastric contraction were also observed when rats received different types of stressors for many days, with increased *CRF* messenger RNA (mRNA) expression in the paraventricular nucleus (PVN) of the hypothalamus, which resulted in increased hypothalamus-pituitary-adrenal (HPA) axis activities (Zheng et al., 2010). Although peripheral CRF receptor antagonists have been developed, the efficiency of the antagonists to treat stress-induced GI dysmotility remains to be further investigated (Kormos and Gaszner, 2013; Bali et al., 2014).

Acupuncture has widely been used to treat numerous diseases for over 3,000 years across Asia and has most recently gained popularity as an alternative or complementary treatment in Western cultures (Campbell, 2002; Hurtak, 2002). According to the World Health Organization (2003), acupuncture is useful as adjunct therapy in more than 50 disorders. In particular, acupuncture and electroacupuncture (EA) at acupoint Zusanli, stomach 36 (ST-36) has been used in patients for a variety of conditions including GI diseases (Hurtak, 2002). ST-36 is one of the most commonly used acupoints for FGIDs, including FD and irritable bowel syndrome (IBS) (Chan et al., 1997; Diehl, 1999; Fireman et al., 2001). Studies have shown that EA at ST-36 can attenuate the HPA axis activity and sympathetic nervous system (SNS) pathway, and in turn reduce the stress responses in patients and rats (Middlekauff et al., 2002; Yang et al., 2002; Eshkevari et al., 2013; Zhou et al., 2017). Interestingly, recent studies also found that EA can increases hypothalamic NPY expression and reduce the stress responses in chronic stress conditions (Lee et al., 2009; Sun et al., 2015). Neuropeptide Y (NPY), belongs to the pancreatic polypeptide family, a 36 amino acids peptide, which is expressed at high levels within the mammalian nervous system (Adrian et al., 1983; Allen et al., 1983). NPY is also synthesized and released from peripheral sympathetic neurons (Heilig and Thorsell, 2002; Heilig, 2004). Several studies have shown that NPY is involved in stress related disorders, such as depression, anxiety, and post-traumatic stress disorder (PTSD) (Rasmusson et al., 2010; Sabban et al., 2016).

Neuropeptide Y inhibits the biological actions of CRF and is involved in the termination of the stress and anxiety response (Morales-Medina et al., 2010). Endogenous NPY is potently anxiolytic, acting as a buffer that promotes behavioral adaptation to cope with stress (Primeaux et al., 2005; Reichmann and Holzer, 2016). The actions of NPY are mediated through at least five G protein coupled receptors (Dumont et al., 1998). The anxiolytic effect of NPY is mediated primarily through the Y1 receptor (Morales-Medina et al., 2010; Reichmann and Holzer, 2016). Our recent study also showed that the central NPY *via* the Y1 receptor plays an important role in mediating the adaptation mechanism against repeated restraint stress in rats (Yang et al., 2018).

Furthermore, in the study of the inhibition mechanism of NPY on CRF, it was found that central NPY could regulate the excitability of the central nuclei of the amygdala (CeA) through the gamma-aminobutyric acid (GABA)_*A*_ receptor and improves the adaptability of organisms to stress responses (Molosh et al., 2013). In addition, NPY and GABA were co-expressed in the arcuate nucleus of the hypothalamus (ARC) neurons and projected to PVN (Muroya et al., 2005). The regulation of NPY on the feeding function is also mediated by the GABA_*A*_ receptor (Pu et al., 1999). Therefore, it is necessary to further explore the regulatory and inhibitory mechanism of NPY on CRF through the GABA_*A*_ receptor under chronic stress.

The present study aimed to evaluate, using a CCS rat model, whether EA at ST-36 could up-regulate NPY system activity, and in turn attenuate the HPA axis activity in CCS conditions. It was also evaluated whether their effects help to restore GI dysmotility. The inhibitory effect of NPY on CRF through the GABA_*A*_ receptor system was further explored, to clarify the mechanisms of stimulatory effects of EA on GI dysmotility.

## Materials and Methods

### Ethics Statement

All research procedures were carried out in accordance with the guidelines for the ethical review of laboratory animal welfare People’s Republic of China National Standard GB/T 35892-2018 and approved by the Animal Care and Use Committee of China Medical University. Efforts were made to minimize animal suffering and the number of animals used.

### Animals

Male Sprague-Dawley rats (adult, 260–300 g, obtained from the laboratory animal center of China Medical University) were housed in individual cages under conditions (22–24°C, 12:12 h light/dark cycle) with *ad libitum* access to food and water. All experiments were started at 9 AM each day. The approval reference number is CMU2019225.

### Chronic Complicated Stress Loading

The rats (*n* = 6–8) received different types of stressors for 7 consecutive days, as previously reported (Zheng et al., 2010; Yang et al., 2019). The stress paradigms used were fasten restraint stress (FRS), force swimming stress (FSS), cold restraint stress (CRS), and water avoidance stress (WAS). The specific conditions for each type of stress are as follows:

(1) FRS: rats were placed on a wooden plate with their trunks wrapped in a confining harness for 90 min. For the control group (*n* = 6–8), the rats were housed in original individual cages for 90 min, but were given limited access to food and water.

(2) FSS: rats were placed individually in a plastic tank (52 × 37 × 20 cm) filled with room temperature (RT) water to a depth of 15 cm for 20 min. The depth of the water forced the animal to swim or float without their hindlimbs touching the bottom of the tank. Control rats were placed individually in a waterless container tank for 20 min.

(3) CRS: rats were kept restrained at 4°C for 45 min. Control rats were kept at RT for 45 min.

(4) WAS: rats were placed on a platform (6 × 8 cm) in the middle of a plastic container (50 × 30 × 20 cm) filled with RT water to 1 cm below the height of the platform for 60 min. Control rats were placed on the same platform in a waterless container for 60 min.

Rats were exposed to different stressors each day for 7 days:

(1) Day 1: FRS [90 min, ante meridiem (AM)], FSS [20 min, post meridiem (PM)]; Day 2: CRS (45 min, AM); Day 3: FRS (90 min, AM), WAS (60 min); Day 4: CRS (45 min, AM); Day 5: FRS (90 min, AM), FSS (20 min, PM); Day 6: CRS (45 min, AM); Day 7: FRS (90 min, AM).

### Electro-Acupuncture Procedure

The electro-acupuncture (EA) ST-36 in a rat is located at the proximal one-fifth of the craniolateral surface of the leg distal to the head of the tibia, in a depression between the muscles of the cranial tibia and long digital extensor, bilaterally. These points were identified using rat mapping for acupuncture, as previously reported (Iwa et al., 2006). To avoid the spontaneous removal of inserted electrodes from the rat body, thin stainless steel needles 34G (0.22 mm) and 1 inch (25 mm) (Millennia, Shanghai, China) were bent in a hook shape, as previously reported (Iwa et al., 2006; Yoshimoto et al., 2012). Bilaterally inserted needles at the hind limbs were connected to an EA machine (ITO-ES320, Japan) and stimulated by electricity (10 Hz, 3 V, 0.5 ms) for 30 min. EA was performed every day prior to the stress loading.

The sham group received non-acupuncture map points EA treatment on the back of the animal (EA at back), 2 cm lateral to the tail region, which was then connected to the EA machine and stimulated by electricity for 30 min, every day prior to the stress loading.

To exclude the influence of anesthesia and restraint conditions, rats were allowed to move freely in the cage during the EA procedure, as reported previously (Iwa et al., 2006; Yoshimoto et al., 2012). The performance of the rats was monitored, and the wires connecting with the inserted needles were fixed on the back by tape to prevent the researchers from being bitten.

### Blood Collection and Hormone Assays

The experimental rats were euthanized immediately by pentobarbital sodium (Sigma-Aldrich, St. Louis, MO, United States, 200 mg/kg intraperitoneal injection) after FRS, at the 7th day of CCS loading.

At the time of death, trunk blood was collected immediately *via* a cardiac puncture, and then the samples were allowed to clot in tubes and were centrifuged at 4°C for 10 min at 3,000 rpm to separate the serum. The serum fraction was stored at −80°C for further analysis. Corticosterone concentrations were measured by enzyme-linked immunosorbent assay (ELISA) using a corticosterone ELISA kit (Cat# ADI-900-097, Enzo Life Sciences, Plymouth meeting, PA, United States, detection level 32–20,000 pg/ml, sensitivity 27.0 pg/ml), as previously reported (Kinn Rød et al., 2017). NPY concentrations were measured by NPY EIA kit (Cat**#** EK-049-03, Phoenix Pharmaceuticals, Belmont, CA, United States, detection level 0.09–1.43 ng/ml, sensitivity 0.09 ng/ml), as previously reported (Serova et al., 2013; Yang et al., 2018). All procedures were carried out according to the manufacturer’s instructions. The experiments were run in triplicate and the results represent their average values.

### Quantitative Real-Time Polymerase Chain Reaction

The rat brain tissue micropunching technique was applied to acquire hypothalamus tissue samples from specific regions with micro-punchers. Briefly, after stress loading, the experimental rats were euthanized, and the brains were removed immediately and cut into 450 μm coronal sections. Punches were collected from the left and right PVN (1.8 mm caudal to bregma; 0.4 mm lateral to midline; 7.6 mm ventral to the brain surface), as previously reported (Zheng et al., 2010; Yang et al., 2018). All coordinates were based on the rat brain atlas and hypothalamic images previously reported (Paxinos and Watson, 1997).

Samples were stored at −80°C until use. Total RNA was extracted from the brain tissues using Trizol (Invitrogen, Carlsbad, CA, United States), and trace DNA contamination was removed by DNase digestion (Promega, Madison, WI, United States). Complementary DNA was synthesized from 3 μg total RNA by use of Superscript III reverse transcriptase (Invitrogen, Carlsbad, CA, United States).

The following primers were designed to amplify rat CRF: sense primer 5′-CCAGGGCAGAGCAGTTAGCT-3′, antisense primer 5′-CAAGCGCAACATTTCATTTCC-3′. The following primers were designed to amplify rat NPY: sense primer 5′-CAGAGGCGCCCAGAGCAG-3′, antisense primer 5′-CAGCCCCATTCGTTTGTTACC-3′. For an internal control, the following primers were designed to amplify rat β–actin: sense primer 5′-TGGCACCACACCTTCTACAATGAG-3′, antisense primer 5′-GGGTCATCTTTTCACGGTTGG-3′, as previously reported (Zheng et al., 2010; Yang et al., 2018).

Quantitative polymerase chain reaction (PCR) was performed using SYBR Premix Ex Taq (TaKaRa Biotech, Dalian, China). Amplification reactions were performed using the ABI 7500 Real-time PCR instrument (Applied Biosystems, San Mateo, CA, United States). Initial template denaturation was performed for 30 s at 95°C. The cycle profiles were programmed as follows: 5 s at 95°C (denaturation), 20 s at 60°C (annealing), and 15 s at 72°C (extension). Forty-five cycles of the profile were run, and the final cooling step was continued for 30 s at 40°C. Quantitative measurement of each mRNA sample was achieved by establishing a linear amplification curve from serial dilutions of each plasmid containing the amplicon sequence. Amplicon size and specificity were confirmed by melting curve analysis. The relative amount of each mRNA was normalized by the amount of β-*actin* mRNA, as previously reported (Zheng et al., 2010; Yang et al., 2018).

### ICV Cannulation and Administration of GABA_*A*_ Receptor Antagonist

The rats were anesthetized with isoflurane (5% for induction; 2% for maintenance in pure oxygen gas; RWD life science, Shenzhen, China), and placed in a stereotaxic apparatus (RWD life science, Shenzhen, China). After the skin and muscles of the head were dissected, a 24-gage plastic sterile cannula (Plastics One, United States) was implanted into the right lateral ventricle (1.5 mm caudal, 2 mm lateral from the bregma; 6 mm ventral from the skull surface), as previously reported (Zheng et al., 2010; Yang et al., 2018). The cannula was fixed with cement (Kyowa, Tokyo, Japan) and acrylic resin (Shofu, San Marcos, CA, United States). A stainless-steel obturator (Plastics One, Roanoke, VA, United States), was inserted to maintain cannula patency. After cannulation, the rats were allowed to recover for 1 week.

To investigate whether the GABA_*A*_ receptor is involved in NPY mediated restoration following CCS, Bicuculline Methiodide (BMI, Sigma Aldrich, MO, United States) (100 ng/5 μl, ICV) was injected daily, 15 min prior to the stress loading. Saline (5 μl, ICV) was injected daily for the controls. It has been shown that ICV administration of BMI (100 ng) was effective in the antagonization of GABA_*A*_ receptor subtypes in rats, as previously reported (Li et al., 2006; Bülbül et al., 2011).

At the end of the experiment, the implantation site of the ICV-cannula was confirmed by the presence of Evans blue (Sigma-Aldrich, St. Louis, MO, United States, 5%; 1 μl) after injection *via* the cannula, as previously reported (Zheng et al., 2010; Yang et al., 2018).

### Monitoring of Gastric Motility

Rats were anesthetized with isoflurane (2%). Strain gage transducers were implanted on the antrum to record gastric contractions. All wires were tunneled subcutaneously to exit at the back of the rat’s neck and were protected by a protective jacket (Star Medical, Tokyo, Japan). The abdominal wall was closed, and the rats were housed individually with access to a standard diet and tap water. After 7 days, the rats completely recovered from the surgery, including their body weight and daily food intake. Rats on a fixed-feeding schedule (food administered 22-6 PM daily) were monitored from 8 AM to 4 PM for gastric motility, as previously reported (Zheng et al., 2010; Yang et al., 2018): The wires from the transducers were connected to a recording system (Power Lab 8SP; AD Instruments, Colorado Springs, CO, United States). Gastric contractions were monitored before, during, and after restraint stress. Quantification of gastric motility was studied by the calculating motility index (MI).

MI was equivalent to the area under the curve of the motility recording.

The MI was defined as MI log e (sum of amplitudes × total number of contraction waves + 1) which is equivalent to the area under the contractility recording curve and the baseline. MI was calculated using a computer-assisted system (Power Lab; AD Instruments, Colorado Springs, CO, United States), as previously reported (Zheng et al., 2010; Yang et al., 2018).

### Measurement of Solid Gastric Emptying

Rats were fasted for 24 h prior to the measurement of GE. Preweighed standard rodent pellets (1.6 g) were given, and the rats that did not consume 1.6 g of food within 10 min were excluded from the study. After feeding, in the control group, rats were put back into their original cages for 90 min, but with limited access to food and water. The rats were then euthanized by pentobarbital sodium. Immediately after finishing the feeding, the rats in the stress group were subjected to restraint stress for 90 min. The experimental rats were then euthanized as mentioned above. The stomach was surgically isolated and removed. The gastric contents were recovered from the stomach, dried, and weighed. Solid GE was calculated according to the following formula, as previously described (Zheng et al., 2010; Yang et al., 2018):

GE(%)=[1-(driedweightoffoodrecoveredfromstomach/weightoffoodintake)]×100

### Experimental Design

#### Experiment 1

To study the effects of EA at ST-36 in the CCS rat model, EA was performed every day prior to the stress loading. Non-acupuncture map points with EA treatment on the back of the animal (EA at back) made up the sham group, and NS (non-stressed) rats were the controls. Central *CRF* and *NPY* mRNA expression (in PVN) were analyzed, serum corticosterone and NPY concentrations were measured, gastric motor function was evaluated by a motility recording system, and GE was measured.

#### Experiment 2

The inhibitory mechanism of NPY on CRF *via* the GABA_*A*_ receptor under the CCS condition was studied. In ICV cannulated rats, the GABA_*A*_ receptor antagonist BMI was injected daily, 15 min prior to EA at ST-36. Non-acupuncture map points on the back of the rats (EA at back), made up the sham group. The rats were then subjected to the CCS stress loading, and in NS rats, saline (5 μl, ICV) was injected daily as a control. Central *CRF* and *NPY* mRNA expression (in PVN) were analyzed, serum corticosterone and NPY concentrations were measured, gastric motor function was evaluated by motility recording system, and GE was measured.

### Statistical Analysis

An analysis was performed using SPSS 20.0 statistical software (SPSS Inc., Chicago, IL, United States). Results were shown as mean ± standard error (SEM). Statistical analyses were performed using a two-way classification ANOVA with Tukey’s *post hoc* tests to determine the significant interaction between different stress groups and drug treatment. Differences with *P* < 0.05 were considered statistically significant.

## Results

### Experiment 1

#### Effects of EA at ST-36 on Central *CRF* and *NPY* mRNA Expression in Response to Chronic Complicated Stress

**FIGURE 1:**
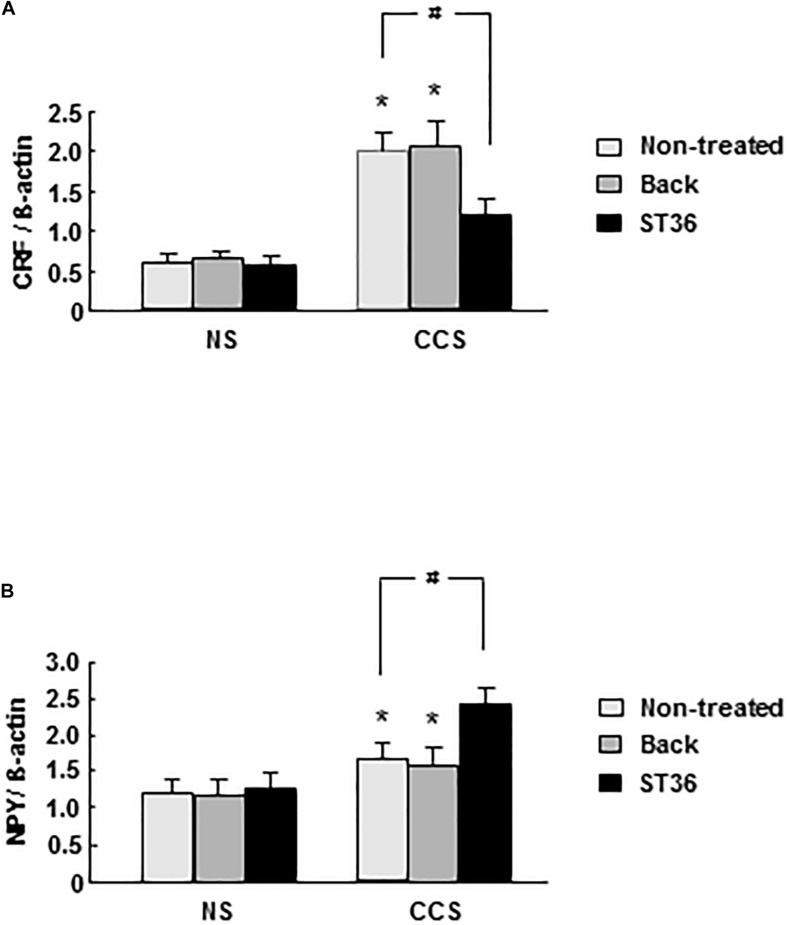
Effects of EA on central *CRF* **(A)** and *NPY* **(B)** mRNA expression in response to CCS. **(A)** In the non-stressed (NS) groups, both EA at back and at ST-36 had no effect on the *CRF* mRNA expression. In the CCS groups, *CRF* mRNA expression increased significantly (non-treated). EA at back did not change the *CRF* mRNA expression significantly, however, the EA at ST-36 significantly decreased the *CRF* mRNA expression. **(B)** In the NS groups, both EA at back and at ST-36 had no effect on the *NPY* mRNA expression. In the CCS groups, *NPY* mRNA expression increased significantly. EA at back did not change the *CRF* mRNA expression significantly, however, the EA at ST-36 further significantly increased the *NPY* mRNA expression. The mRNA expression was standardized with the ratio of internal control of β-actin (*n* = 6, *P < 0.05 compared with NS non-treated group, ^#^*P* < 0.05 compared with CCS non-treated group).

In the NS groups, *CRF* mRNA expression in the PVN showed very low levels. Both the sham group (EA at back) and EA at ST-36 did not change the *CRF* mRNA expression significantly. In the CCS groups, *CRF* mRNA expression increased significantly compared to that of NS rats (*n* = 6). The sham group (EA at back) did not change the *CRF* mRNA expression significantly, however, the EA at ST-36 significantly decreased the *CRF* mRNA expression compared to that of the CCS non-treated group (*n* = 6, *P* < 0.05, Figure 1A).

In the NS groups, *NPY* mRNA expression in the PVN showed low levels. Both the sham group (EA at back) and EA at ST-36 did not change the *NPY* mRNA expression significantly. In the CCS groups, *NPY* mRNA expression increased significantly compared to that of NS rats (*n* = 6). The sham group (EA at back) did not change the *NPY* mRNA expression significantly, however, the EA at ST-36 further significantly increased the *NPY* mRNA expression compared to that of the CCS non-treated group (*n* = 6, *P* < 0.05, Figure 1B).

### Effects of EA at ST-36 on Serum Corticosterone and NPY Levels in Response to Chronic Complicated Stress

**FIGURE 2:**
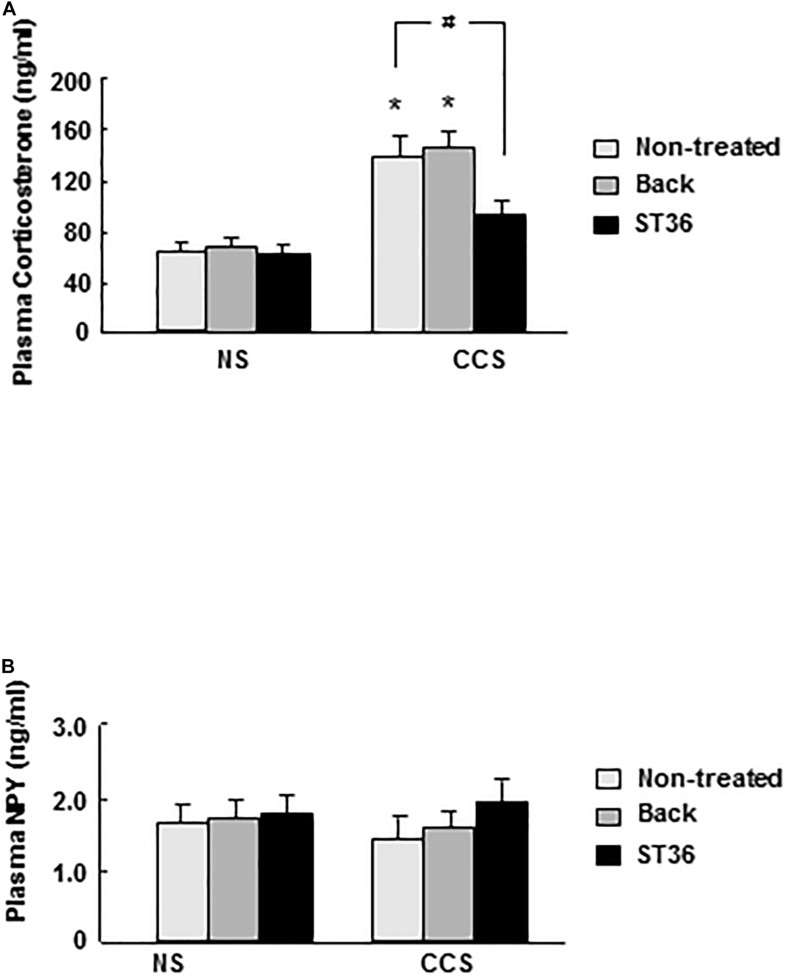
Effects of EA on serum corticosterone **(A)** and NPY **(B)** levels in response to CCS. **(A)** In the non-stressed (NS) groups, both EA at back and at ST-36 had no effect on the corticosterone level. In the CCS groups, corticosterone level increased significantly (non-treated). EA at back did not change the corticosterone level significantly, however, EA at ST-36 significantly decreased the corticosterone level. **(B)** In the non-stressed (NS) groups, both EA at back and at ST-36 had no effect on the NPY level. In the CCS groups, NPY level decreased, but not shown significantly changed (non-treated). Both EA at back and EA at ST-36 also did not change the NPY level significantly (*n* = 6, *P < 0.05 compared with NS non-treated group, ^#^*P* < 0.05 compared with CCS non-treated group).

In the NS groups, serum corticosterone concentration showed low levels (63.6 ± 6.6 ng/ml, *n* = 6). Both the sham group (EA at back) and EA at ST-36 did not change the corticosterone level significantly (66.5 ± 7.3 and 62.8 ± 7.5 ng/ml, respectively, *n* = 6). In the CCS groups, serum corticosterone concentration significantly increased to 135.4 ± 13.6 ng/ml, compared to that of NS rats (*n* = 6, *P* < 0.025). The sham group (EA at back) did not change the corticosterone level significantly (143.3 ± 12.5 ng/ml, *n* = 6). However, the EA at ST-36 significantly decreased the corticosterone level to 89.7 ± 9.6 ng/ml, compared to that of CCS non-treated rats (*n* = 6, *P* < 0.05, Figure 2A).

In the NS groups, serum NPY concentration showed basal levels (1.73 ± 0.13 ng/ml, *n* = 6). Both the sham group (EA at back) and EA at ST-36 did not change the NPY level significantly (1.78 ± 0.13 and 1.83 ± 0.15 ng, respectively, *n* = 6). In the CCS groups, serum NPY level decreased to 1.43 ± 0.18 ng/ml but did not show significant change (*n* = 6). The sham group (EA at back) did not change the serum NPY level significantly (1.69 ± 0.11 ng/ml, *n* = 6). The EA at ST-36 also did not change the serum NPY level significantly (1.99 ± 0.23 ng/ml, *n* = 6) compared to that of the CCS non-treated group (*n* = 6, *P* < 0.05, Figure 2B).

#### Effects of EA at ST-36 on Gastric Motility Recording, MI Changes and Gastric Emptying in Response to CCS

**FIGURE 3:**
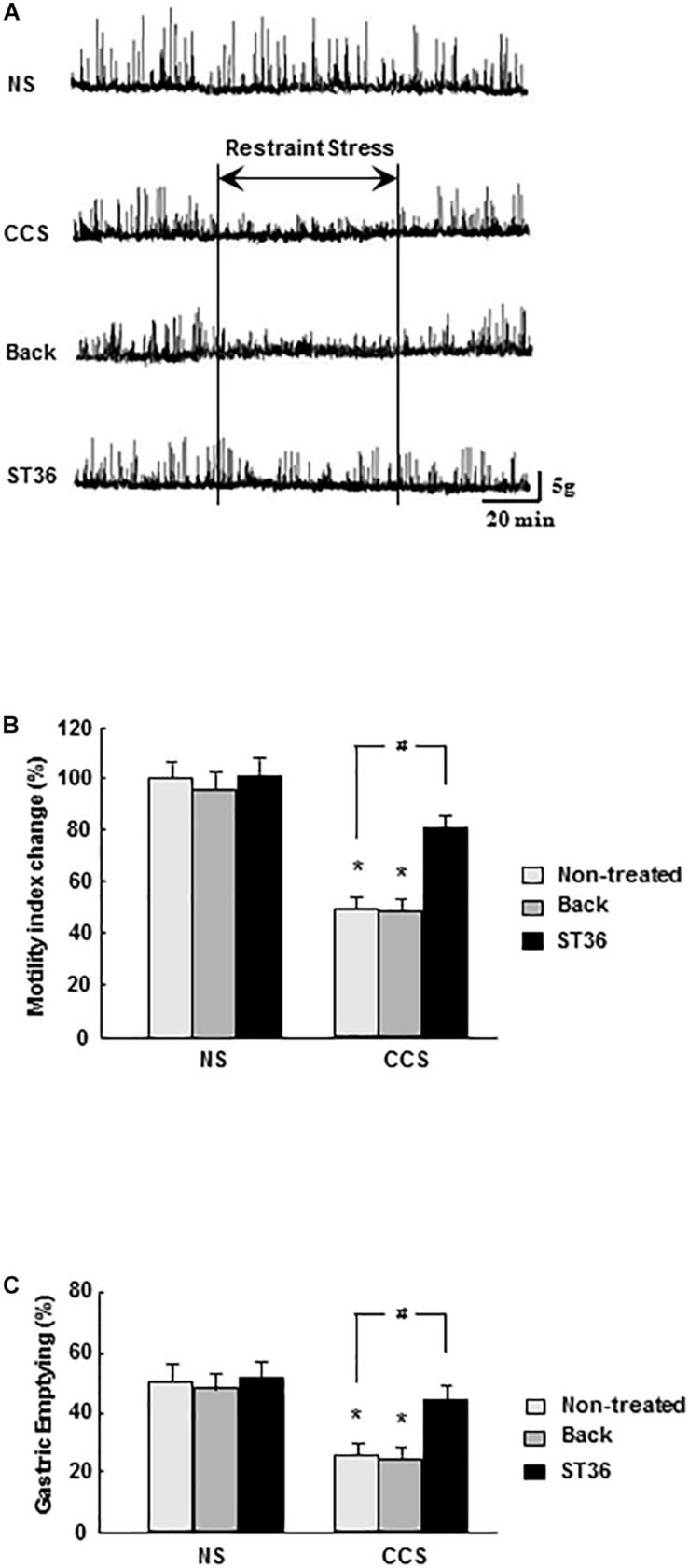
Effects of EA on gastric motility **(A)**, gastric MI changes **(B)**, and GE **(C)** in response to CCS. **(A)** The gastric phase III-like contractions in the non-stressed groups (NS). EA at back and EA at ST-36 had no effect on the gastric contractions (data not shown). CCS abolished gastric phase III-like contractions in the non-treated group. EA at back had no effects on the gastric contractions. However, the EA at ST-36 helped to partially restore the gastric phase III-like contractions. **(B)** In the NS group, EA at back and EA at ST-36 did not significantly alter the gastric MI change. In the CCS groups, gastric MI change was significantly decreased (non-treated). EA at back did not alter the gastric MI change significantly, however, the EA at ST-36 significantly increased the MI change. **(C)** In the NS groups, EA at back and EA at ST-36 did not significantly change the GE. In the CCS groups, the GE was significantly decreased. EA at back did not change the GE significantly, however, the EA at ST-36 significantly increased the GE (*n* = 6, *P < 0.05 compared with NS non-treated group, ^#^*P* < 0.05 compared with CCS non-treated group).

In the NS groups, regular gastric phase III-like contractions were observed in fixed-fed rats. Both the sham group (EA at back) and EA at ST-36 had no effects on the amplitude and frequency of gastric phase III-like contractions (data not shown). In the CCS groups, restraint stress abolished gastric phase III-like contractions. The sham group (EA at back) had no effects on the amplitude and frequency of gastric phase III-like contractions. However, the EA at ST-36 helped to partially restore the gastric phase III-like contractions in the CCS groups (Figure 3A).

Each recorded experiment was individually repeated at least three times and similar results were obtained (*n* = 4).

In the NS groups, the gastric MI change was 100 ± 8% (n = 6). Both the sham group (EA at back) and EA at ST-36 did not alter the gastric MI change significantly (96 ± 8% and 102 ± 6%, respectively, n = 6). In the CCS groups, the gastric MI change was significantly decreased to 51 ± 5% (*P* < 0.05, n = 6). The sham group (EA at back) did not significantly change the gastric MI change (49 ± 5%, n = 6). However, the EA at ST-36 significantly helped to restore the gastric MI change to 80 ± 5% (*P* < 0.05, n = 6, Figure 3B).

In the NS groups, the GE was 51.4 ± 2.9% (n = 6). Both the sham group (EA at back) and EA at ST-36 did not alter the GE significantly (48.6 ± 2.9% and 53.4 ± 3.5%, respectively, n = 6). In the CCS groups, the GE was significantly decreased to 25.4 ± 2.2% (*P* < 0.05, n = 6). The sham group (EA at back) did not significantly change GE (24.7 ± 2.5%, n = 6), however, the EA at ST-36 significantly increased the GE to 45.9 ± 2.8% (*P* < 0.05, n = 6, Figure 3C).

## Experiment 2

### Effects of EA at ST-36 and GABA_*A*_ Receptor Antagonist BMI on Central *CRF* mRNA Expression and *NPY* mRNA Expression in Response to CCS

**FIGURE 4:**
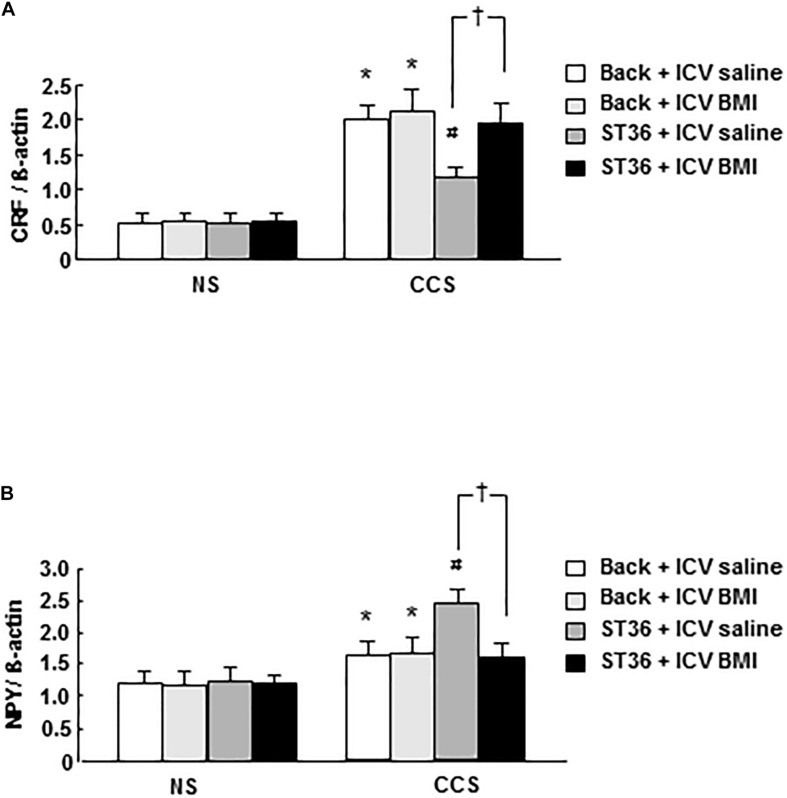
Effects of EA at ST-36 and GABA_*A*_ receptor antagonist on central *CRF* mRNA expression **(A)** and *NPY* mRNA expression **(B)** in response to CCS. **(A)** In the NS groups, *CRF* mRNA expression showed low level, EA at back (with ICV administered saline or BMI) did not change the *CRF* mRNA expression significantly. However, EA at ST-36 (with ICV administered saline or BMI) also did not change the *CRF* mRNA expression significantly. In the CCS conditions, CCS highly elevated the *CRF* mRNA expression in the sham group (EA at back, and saline ICV administered). In the EA at back groups, ICV administered BMI did not change the *CRF* mRNA expression significantly. However, in the EA at ST-36 groups, the reduced *CRF* mRNA expression was significantly increased by ICV administered BMI. **(B)** In the NS groups, *NPY* mRNA expression showed low level, EA at back (with ICV administered saline or BMI) did not change the *NPY* mRNA expression significantly. However, EA at ST-36 (with ICV administered saline or BMI) also did not change the *NPY* mRNA expression significantly. In the CCS conditions, CCS elevated the *NPY* mRNA expression in the sham group (EA at back, and saline ICV administered). In the EA at back groups, ICV administered BMI did not change the *NPY* mRNA expression significantly. However, in the EA at ST-36 groups, the further significantly increased *NPY* mRNA expression (saline ICV administered), was significantly inhibited by ICV administered BMI (*n* = 6, *P < 0.05 compared with NS non-treated group, ^#^*P* < 0.05 compared with CCS non-treated group, ^†^*P* < 0.05 compared with CCS ST36 + ICV saline group).

In the NS groups, *CRF* mRNA expression showed low levels (EA at back, and saline 5 μl ICV administered as a control), however, the combination of EA at back or ST-36 with ICV administered saline or BMI did not change the *CRF* mRNA expression significantly (*n* = 6). In the CCS conditions, the *CRF* mRNA expression in the sham group (EA at back, and saline ICV administered) was significantly elevated. ICV administered BMI (100 ng/5 μl, 15 min prior to stress the loading for 7 consecutive days) did not change the *CRF* mRNA expression significantly (*n* = 6). However, in the EA at ST-36 groups, the decreased *CRF* mRNA expression was significantly increased by ICV administered BMI (*n* = 6, *P* < 0.05, saline 5 μl ICV injected as a control; Figure 4A).

In the NS groups, *NPY* mRNA expression showed low level (EA at back, and saline 5 μl ICV administered as a control), however, the combination of EA at back or ST-36 with ICV administered saline or BMI did not change the *NPY* mRNA expression significantly (*n* = 6). In the CCS conditions, the *NPY* mRNA expression increased significantly in the sham group (EA at back, and saline ICV administered). ICV administered BMI (EA at back) did not change the *NPY* mRNA expression significantly (*n* = 6). However, in the EA at ST-36 groups, the further significantly increased *NPY* mRNA expression (saline ICV administered), was significantly inhibited by ICV administered BMI (*n* = 6, *P* < 0.05, saline 5 μl ICV injected as a control; Figure 4B).

### Effects of EA at ST-36 and GABA_*A*_ Receptor Antagonist BMI on Serum Corticosterone and NPY Levels in Response to CCS

**FIGURE 5:**
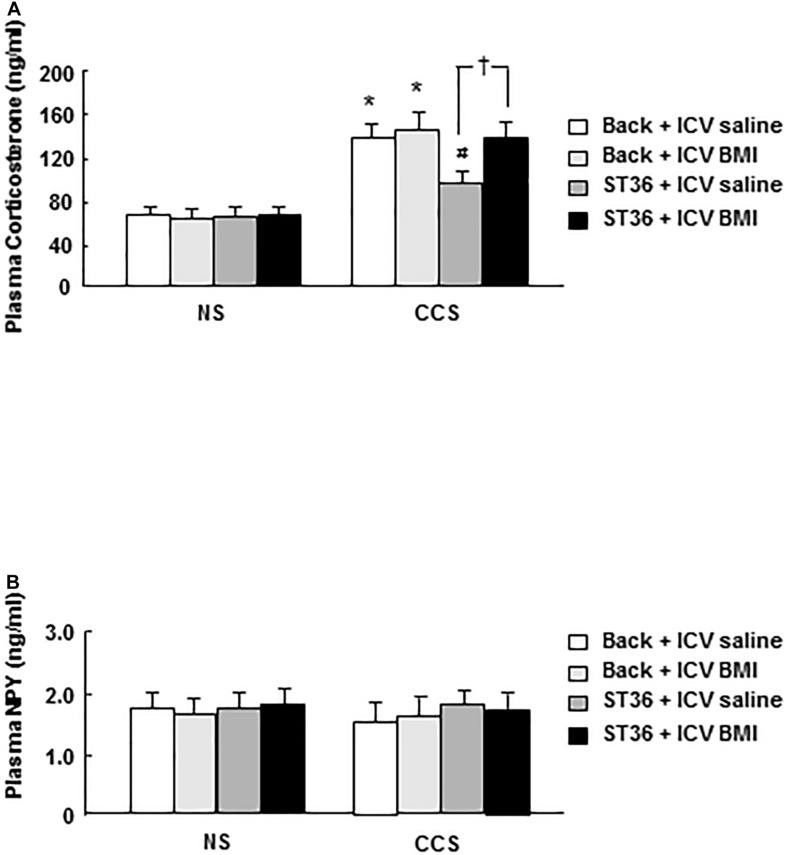
Effects of EA at ST-36 and GABA_*A*_ receptor antagonist on serum corticosterone **(A)** and NPY **(B)** levels in response to CCS. **(A)** In the NS groups, serum corticosterone concentration showed low level, EA at back (with ICV administered saline or BMI) did not change the corticosterone level significantly. However, EA at ST-36 (with ICV administered saline or BMI) also did not change the corticosterone level significantly. In the CCS conditions, CCS highly elevated the serum corticosterone level in the sham group (EA at back, and saline ICV administered). In the EA at back groups, ICV administered BMI did not change the corticosterone level significantly. However, in the EA at ST-36 groups, the significantly decreased corticosterone level was completely antagonized by ICV administered BMI. **(B)** In the NS groups, serum NPY concentration showed low level, EA at back (with ICV administered saline or BMI) did not change the NPY level significantly. However, EA at ST-36 (with ICV administered saline or BMI) also did not change the NPY level significantly. In the CCS conditions, the serum NPY level in the sham group did not show significant change. In the EA at back groups, ICV administered BMI did not change the NPY level significantly. However, EA at ST-36 (with ICV administered saline or BMI) also did not change the NPY level significantly (*n* = 6, *P < 0.05 compared with NS non-treated group, ^#^*P* < 0.05 compared with CCS non-treated group, ^†^*P* < 0.05 compared with CCS ST36 + ICV saline group).

In the NS groups, the serum corticosterone concentration showed low levels (64.2 ± 7.0 ng/ml, EA at back, and saline 5 μl ICV administered as a control), however, the combination of EA at back or ST-36 with ICV administered saline or BMI did not change the corticosterone concentration significantly (62.4 ± 8.1, 63.9 ± 6.9, and 65.6 ± 6.7, respectively, *n* = 6). In the CCS conditions, the serum corticosterone concentration in the sham group (EA at back, and saline ICV administered) was significantly elevated (139.9 ± 10.4 ng/ml, *n* = 6). ICV administered GABA_*A*_ receptor antagonist BMI did not change the serum corticosterone concentration significantly (143.8 ± 12.4 ng/ml, *n* = 6, saline 5 μl ICV injected as a control). However, in the EA at ST-36 groups, the decreased serum corticosterone level was significantly increased by ICV administered BMI (from 98.9 ± 9.7 to 137.7 ± 10.2 ng/ml, *n* = 6, *P* < 0.05, saline 5 μl ICV injected as a control; Figure 5A).

In the NS groups, the serum NPY concentration showed low levels (1.79 ± 0.14 ng/ml, EA at back, and saline 5 μl ICV administered as a control), however, the combination of EA at back or ST-36 with ICV administered saline or BMI did not change the NPY concentration significantly (1.75 ± 0.13, 1.80 ± 0.12, and 1.83 ± 0.15 ng/ml, respectively, *n* = 6). In the CCS conditions, the serum NPY level in the sham group decreased to 1.60 ± 0.14 ng/ml but did not show a significant change (*n* = 6). However, the combination of EA at back or ST-36 with ICV administered saline or BMI did not change the NPY concentration significantly (1.65 ± 0.18, 1.86 ± 0.15, and 1.79 ± 0.13 ng/ml, respectively, *n* = 6, saline 5 μl ICV injected as a control; Figure 5B).

### Effects of EA at ST-36 and GABA_*A*_ Receptor Antagonist BMI on Gastric Motility Recording, Motility Index (MI) Changes and Gastric Emptying in Response to CCS

**FIGURE 6:**
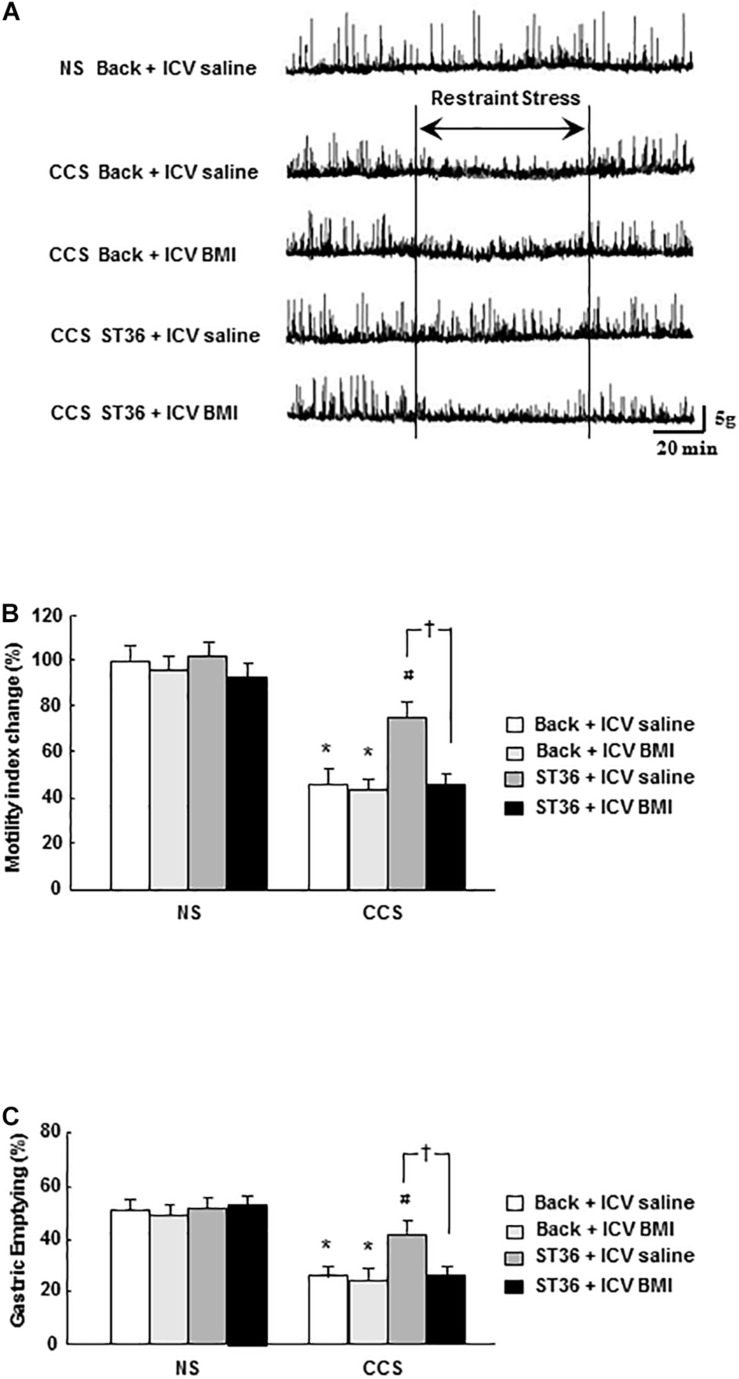
Effects of EA at ST-36 and GABA_*A*_ receptor antagonist on gastric motility **(A)**, gastric MI change **(B)**, and GE **(C)** in response to CCS. **(A)** The gastric phase III-like contractions in the non-stressed groups (NS). EA at back (with ICV administered saline or BMI) and EA at ST-36 (with ICV administered saline or BMI) both had no effects on gastric contractions (data not shown). In the CCS conditions, CCS abolished gastric phase III-like contractions in the sham group (EA at back, and saline ICV administered). ICV injection of BMI also had no effect on the gastric contractions. However, in the EA at ST-36 groups, the partially restored gastric contraction was completely abolished by ICV administered of BMI. **(B)** In the NS groups, the gastric MI change showed high value. EA at back (with ICV administered saline or BMI) had no effects on the gastric MI change. However, EA at ST-36 (with ICV administered saline or BMI) also had no impacts on the gastric MI change. In the CCS conditions, CCS significantly decreased the gastric MI change in the sham group (EA at back, and saline ICV administered). ICV injection of BMI also had no effect on the gastric MI change. However, in the EA at ST-36 groups, the significantly increased gastric MI change was completely antagonized by ICV administered BMI. **(C)** In the NS groups, the GE showed high value. EA at back (with ICV administered saline or BMI) had no effects on the GE. However, EA at ST-36 (with ICV administered saline or BMI) also had no impacts on the GE. In the CCS conditions, CCS significantly decreased the GE in the sham group (EA at back, and saline ICV administered). ICV injection of BMI also had no effect on the GE. However, in the EA at ST-36 groups, the significantly increased GE was completely antagonized by ICV administered BMI (*n* = 6, *P < 0.05 compared with NS non-treated group, ^#^*P* < 0.05 compared with CCS non-treated group, ^†^*P* < 0.05 compared with CCS ST36 + ICV saline group).

In the NS groups, regular gastric phase III-like contractions were observed in fixed-fed rats. The combination of EA at back or ST-36 with ICV administered saline or BMI had no effects on the amplitude and frequency of gastric phase III-like contractions (data not shown). In the CCS condition, restraint stress abolished gastric phase III-like contractions in the sham group (EA at back, and saline ICV administered). ICV administered GABA_*A*_ receptor antagonist BMI (100 ng/5 μl, 15 min prior to stress the loading for 7 consecutive days) did not change the gastric phase III-like contractions noticeably. However, in the EA at ST-36 groups, the partially restored gastric phase III-like contractions was completely abolished by ICV administered BMI. Each recorded experiment was individually repeated at least three times and similar results were obtained (*n* = 4; Figure 6A).

In the NS groups, the gastric MI change is 100 ± 7% (EA at back, and saline 5 μl ICV administered as a control, *n* = 6). The combination of EA at back or ST-36 with ICV administered saline or BMI did not alter the gastric MI change significantly (97 ± 8, 102 ± 6, and 96 ± 7%, respectively, *n* = 6). In the CCS groups, in the sham group (EA at back, and saline ICV administered), the gastric MI change was decreased to 48 ± 5% (*n* = 6). ICV administered GABA_*A*_ receptor antagonist BMI did not significantly alter the gastric MI change (46 ± 4%, *n* = 6). However, in the EA at ST-36 groups, the partially restored gastric MI change was significantly decreased by ICV administered BMI (from 78 ± 7 to 48 ± 5%, saline 5 μl ICV injected as a control; *n* = 6, *P* < 0.05, Figure 6B).

In the NS groups, the GE was 50.9 ± 3.2% (EA at back, and saline 5 μl ICV administered as a control, *n* = 6). The combination of EA at back or ST-36 with ICV administered saline or BMI did not change the GE significantly (48.4 ± 2.8, 51.1 ± 3.1, and 52.3 ± 2.7%, respectively, *n* = 6). In the CCS groups, in the sham group (EA at back, and saline ICV administered), the GE was decreased to 25.4 ± 2.2% (*n* = 6). ICV administered GABA_*A*_ receptor antagonist BMI did not significantly alter the gastric MI change (24.2 ± 2.1%, *n* = 6). However, in the EA at ST-36 groups, the partially restored GE was significantly decreased by ICV administered BMI (from 43.1 ± 3.8 to 25.2 ± 2.4%, saline 5 μl ICV injected as a control; *n* = 6, *P* < 0.05, Figure 6C).

## Discussion

Restraint stress has been used frequently as a psychogenic and physical stress model in rodents. ARS stimulates the central CRF release and plays an important role in influencing GI motor function (Lenz et al., 1988; Tache and Bonaz, 2007). In contrast to ARS, repeated experiences with the same stressor (chronic repeated restraint stress, CRRS) produce the habituation or diminution of behavioral responses. Our previous studies also shown that in the CRRS condition, the elevated central CRF expression and serum corticosterone level, caused by ARS, was attenuated on day 5 and 7 of consecutive stress loading. ARS-induced GI dysmotility, such as delayed GE or impaired gastric phase III-like contractions, gradually returned to normal levels (Zheng et al., 2009; Yang et al., 2018). As mentioned above, stress adaptation is likely to be an important mechanism in maintaining optimal physiological and psychological functions in the face of repeated stress.

However, in the present study, we set up a CCS model, and found that attenuated gastric motility and delayed GE were also observed when rats received different types of stressors for 7 days, with highly elevated *CRF* mRNA expression in the PVN of the hypothalamus, resulting in activation of the HPA axis. These results are consistent with previous studies (Zheng et al., 2010; Yang et al., 2019).

Previous studies have shown that EA at ST36, can attenuate stress responses acting through not only HPA axis activity but also by stimulating parasympathetic activity and inhibiting the SNS pathway (Middlekauff et al., 2002; Yang et al., 2002; Eshkevari et al., 2013), further resulting in the restoration of impaired gastric motility (Iwa et al., 2006; Zhou et al., 2017). Recent studies also found that EA can increase hypothalamic NPY expression and reduce the stress responses in chronic stress conditions (Lee et al., 2009; Sun et al., 2015).

Neuropeptide Y is one of the most widely expressed neuropeptides in the central nervous system, and its wide distribution suggests its involvement in numerous physiological processes (Heilig and Thorsell, 2002; Heilig, 2004). Endogenous NPY has anxiolytic properties (Hirsch and Zukowska, 2012; Reichmann and Holzer, 2016). NPY might counteract the biological actions of CRF, interact with the HPA axis, and be involved in moderating and improving the body’s ability to cope with stress (Heilig, 2004).

Neuropeptide Y expression in the brain under stress conditions has been well studied, however, the magnitude and the direction of stress-induced NPY alterations heavily depend on the type and duration of the stress. For example, central *NPY* mRNA expression is increased after foot shock stress (Kas et al., 2005; de Lange et al., 2008), but was unaltered by mild stress loading comprising acute water avoidance and acute air puff stress (Ishiguchi et al., 2001; Hassan et al., 2014). However, in ARS, under a moderate degree of stimulus, the *NPY* mRNA expression was still debatable: some studies showed increased central *NPY* mRNA expression (Conrad and McEwen, 2000; Sweerts et al., 2001), and unaltered or decreased results were also reported (Thorsell et al., 1998, 1999). In studies of chronic stress, central NPY expression is usually upregulated in response to repeated stress loading (Sweerts et al., 2001; McDougall et al., 2005). Thus, as mentioned above, central NPY usually reacts to acute stress more than a moderate degree of stress or chronic stress. In our present study, where rats received different types of stressors for 7–9 days, *NPY* mRNA expression increased, and EA at ST36 further up-regulated central NPY and made an adequate termination of the stress responses. These results support and extend the above findings.

Quantitative measurement of CRF and NPY mRNA expression in the PVN of the hypothalamus, is a very effective method, which has been applied in our previous and recent studies (Zheng et al., 2010; Yang et al., 2018, 2019). Efforts were also made to minimize the number of animals used in the current study, and further investigation is needed to examine the protein expression of NPY and CRF in the PVN of the hypothalamus.

In the peripheral nervous system, NPY is found in three main pools: sympathetic nerves, platelets, and the adrenal medulla. The circulating plasma NPY levels are in the low range under resting conditions; however, in many stress conditions, the release of NPY is dependent on the intensity and duration of stress, as well the pattern of sympathetic nerve activation (Hirsch and Zukowska, 2012).

Norepinephrine (NE) is the primary sympathetic neurotransmitter, whereas NPY as a co-transmitter is also released during stress, but the proportions of these two transmitters vary depending on the type of stress. The release of NE is the mildest acute stress; however, NPY requires a more prolonged and/or intense stimulation of sympathetic nerves (Zukowska-Grojec, 1995; Abe et al., 2010).

Our recent study also found that, in a FRS condition, which is a moderate stress stimulus, the serum NPY levels did not increase significantly in response to ARS but were significantly increased at repeated restraint stress (Yang et al., 2018). However, in our present study of the CCS condition, serum NPY levels decreased lower than 1.43 ± 0.18 ng/ml but did not show significant change. This supports the theory that in a higher intensity chronic stress condition, the NPY system will be attenuated and limited, and that central NPY failed to play an important role to produce an adaptation (Zukowska-Grojec, 1995; Hirsch and Zukowska, 2012). Our present study found that EA at ST36 increased the *NPY* mRNA expression and decreased *CRF* mRNA expression at the PVN following CCS, but in the peripheral, EA at ST36 did not change the serum NPY level significantly. Our present results support and extend the recent findings that EA at ST36 probably does not act through the peripheral NPY system but acts through central regulation of the CRF and attenuates the HPA axis (Eshkevari et al., 2015). Further studies are needed to clarify the mechanism that may be involved in the peripheral NPY system.

In experiment 2 of our current study, a further study based on the results of the experiment 1 was performed, to show whether the inhibitory mechanism of NPY on CRF *via* the GABA_*A*_ receptor under CCS condition, in the ICV cannulated CCS rat model, in the GABA_*A*_ receptor antagonist BMI was ICV injected. The sham group was the non-acupuncture map points on the back of the rats (EA at back) as a control, so the non-treated group was not included in experiment 2.

The significance of NPY in stress adaptation seems to be receptor dependent. NPY shows strong affinity for the Y1, Y2, and Y5 receptors (Dumont et al., 1998; Kask et al., 2002; Reichmann and Holzer, 2016). Many studies have demonstrated that the anxiolytic behavioral effect of NPY is mediated primarily through post-synaptic Y1 receptors (Kask et al., 2002; Heilig, 2004). Our previous study also found that central NPY *via* the Y1 receptor plays an important role in mediating the adaptation mechanism against chronic stress (Yang et al., 2018).

However, in the study of the inhibition mechanism of NPY on CRF, it was found that central NPY could regulate the excitability of CeA through the GABA_*A*_ receptor and improved the adaptability of organism to stress responses (Molosh et al., 2013). In addition, NPY and GABA were co-expressed in ARC neurons of the hypothalamus and projected to PVN (Muroya et al., 2005). The regulation of NPY on feeding function is also mediated by the GABA_*A*_ receptor (Pu et al., 1999). GABA is the major inhibitory amino acid transmitter of the mammalian central nervous system. GABA exerts its effects through GABA_*A*_ and GABA_*B*_ receptors. GABA-projecting neurons into the PVN are known to inhibit CRF expression *via* the GABA_*A*_ receptors (Bülbül et al., 2011). Released corticosterone in response to acute stress, inhibits CRF release *via* a feedback mechanism, which is mediated *via* GABA_*A*_ receptors in the PVN (Cullinan et al., 2008). It has been suggested that the GABAergic system is also involved in the inhibitory mechanism of intranasal administration of NPY on *CRF* mRNA expression, in chronic stress conditions.

Our present study found that ICV administered GABA_*A*_ receptor antagonist BMI (100 ng, 15 min prior to the stress loading for 7 consecutive days) did not significantly change the *CRF* mRNA expression, the serum corticosterone level, and the gastric motility in CCS condition. However, in the EA groups, the decreased *CRF* mRNA expression and the serum corticosterone level were significantly antagonized by ICV administered BMI, while the restored gastric contraction and GE was inhibited by BMI. Thus, in a higher intensity chronic stress condition, an EA stimulated up-regulated NPY system was expected in the termination of the stress responses of the CCS condition.

A recent study also found that ICV administered BMI 100 ng (200 pmol) was effective in antagonization of the GABA_*A*_ receptor subtypes in an acute and chronic stress condition, but not in non-stressed rats (Bülbül et al., 2011). In the current study, we also found that ICV administered BMI 100 ng significantly abolished the effects induced by EA, suggesting that the GABAergic system is also involved in the inhibitory mechanism of the up-regulated NPY system induced by EA, in chronic stress conditions. But in non-stressed conditions, administration of BMI did not show significant changes. Our present study agrees with the above report.

However, in non-stressed conditions, the effects of central administration of BMI are still controversial. Previous studies showed that ICV administered BMI (50–200 pmol) significantly increased the central sympathetic neurons and peripheral nerve activities in rats (Li et al., 2006). Conversely, studies also found that central microinjection of BMI (20 pmol) significantly increased the activities of the DMV (dorsal motor nucleus of the vagus) neurons and vagal pathways, and as a consequence produced an increase in gastric motility (Herman et al., 2009). The different results of these studies, may be due to the position, timing, and even the doses of central injection, and further investigation is needed clarify this issue.

## Conclusion

In conclusion, our current study showed that attenuated gastric motor function induced by CCS was significantly restored by EA at ST-36 in rats. EA at ST-36 increased the central *NPY* mRNA expression but not the peripheral NPY level, through central regulation of CRF, and in turn decreased HPA axis activity. The stimulated effect of EA on attenuated gastric motor function was antagonized by ICV injection of the GABA_*A*_ receptor antagonist. These suggest that EA may act on NPY neurons at the hypothalamus, *via* GABA_*A*_ receptor resulting in reduced CRF expression and restoration of gastric dysmotility following CCS.

Our study may contribute to a better understanding of the mechanism and the treatment strategies in GI dysmotility of stress in daily life. GI dysmotility induced by non-habituating stress may be treatable by EA at ST-36, because of its stimulatory effects on the NPY system, which may be a new approach for treatment of stress-induced GI motility disorders.

## Data Availability Statement

All datasets generated for this study are included in the article/supplementary material.

## Ethics Statement

The animal study was reviewed and approved by the Animal Care and Use Committee of China Medical University.

## Author Contributions

YY, HY, RB, and BS performed the experiment. WS and XZ were involved in the study supervision and critical revision of the manuscript. JZ designed the experiment, analyzed the data, and wrote the manuscript. All authors contributed to the article and approved the submitted version.

## Conflict of Interest

The authors declare that the research was conducted in the absence of any commercial or financial relationships that could be construed as a potential conflict of interest.

## References

[B1] Abe K. Kuo L. Zukowska Z. (2010). Neuropeptide Y is a mediator of chronic vascular and metabolic maladaptations to stress and hypernutrition. *Exp. Biol. Med. (Maywood)* 235 1179–1184. 10.1258/ebm.2010.009136 20881322

[B2] Adrian T. E. Allen J. M. Bloom S. R. Ghatei M. A. Rossor M. N. Roberts G. W. (1983). Neuropeptide Y distribution in human brain. *Nature* 306 584–586. 10.1038/306584a0 6358901

[B3] Allen Y. S. Adrian T. E. Allen J. M. Tatemoto K. Crow T. J. Bloom S. R. (1983). Neuropeptide Y distribution in the rat brain. *Science* 221 877–879. 10.1126/science.6136091 6136091

[B4] Bali A. Singh N. Jaggi A. S. (2014). Neuropeptides as therapeutic targets to combat stress-associated behavioral and neuroendocrinological effects. *CNS Neurol. Disord. Drug Targets* 13 347–368. 10.2174/1871527313666140314163920 24625277

[B5] Bülbül M. Babygirija R. Cerjak D. Yoshimoto S. Ludwig K. Takahashi T. (2011). Hypothalamic oxytocin attenuates CRF expression via GABA(A) receptors in rats. *Brain Res.* 1387 39–45. 10.1016/j.brainres.2011.02.091 21382355

[B6] Campbell A. (2002). The origins of acupuncture. *Acupunct. Med.* 20:141. 10.1136/aim.20.2-3.141-a 12216601

[B7] Chan J. Carr I. Mayberry J. F. (1997). The role of acupuncture in the treatment of irritable bowel syndrome: a pilot study. *Hepatogastroenterology* 44 1328–1330. 9356848

[B8] Conrad C. D. McEwen B. S. (2000). Acute stress increases neuropeptide Y mRNA within the arcuate nucleus and hilus of the dentate gyrus. *Brain Res. Mol. Brain Res.* 79 102–109. 10.1016/s0169-328x(00)00105-4 10925147

[B9] Cullinan W. E. Ziegler D. R. Herman J. P. (2008). Functional role of local GABAergic influences on the HPA axis. *Brain Struct. Funct.* 213 63–72. 10.1007/s00429-008-0192-2 18696110

[B10] de Lange R. P. Wiegant V. M. Stam R. (2008). Altered neuropeptide Y and neurokinin messenger RNA expression and receptor binding in stress-sensitised rats. *Brain Res.* 1212 35–47. 10.1016/j.brainres.2008.03.018 18440496

[B11] Diehl D. L. (1999). Acupuncture for gastrointestinal and hepatobiliary disorders. *J. Altern Complement Med.* 5 27–45. 10.1089/acm.1999.5.27 10100029

[B12] Dumont Y. Jacques D. Bouchard P. Quirion R. (1998). Species differences in the expression and distribution of the neuropeptide Y Y1, Y2, Y4, and Y5 receptors in rodents, guinea pig, and primates brains. *J. Comp. Neurol.* 402 372–384. 10.1002/(sici)1096-9861(19981221)402:3<372::aid-cne6>3.0.co;2-2 9853905

[B13] Eshkevari L. Mulroney S. E. Egan R. Lao L. (2015). Effects of acupuncture, RU-486 on the hypothalamic-pituitary-adrenal axis in chronically stressed adult male rats. *Endocrinology* 156 3649–3660. 10.1210/EN.2015-1018 26196540

[B14] Eshkevari L. Permaul E. Mulroney S. E. (2013). Acupuncture blocks cold stress-induced increases in the hypothalamus-pituitary-adrenal axis in the rat. *J. Endocrinol.* 217 95–104. 10.1530/JOE-12-0404 23386059

[B15] Fireman Z. Segal A. Kopelman Y. Sternberg A. Carasso R. (2001). Acupuncture treatment for irritable bowel syndrome. A double-blind controlled study. *Digestion* 64 100–103. 10.1159/000048847 11684823

[B16] Hassan A. M. Jain P. Reichmann F. Mayerhofer R. Farzi A. Schuligoi R. (2014). Repeated predictable stress causes resilience against colitis-induced behavioral changes in mice. *Front. Behav. Neurosci.* 8:386. 10.3389/fnbeh.2014.00386 25414650 PMC4222228

[B17] Heilig M. (2004). The NPY system in stress, anxiety and depression. *Neuropeptides* 38 213–224. 10.1016/j.npep.2004.05.002 15337373

[B18] Heilig M. Thorsell A. (2002). Brain neuropeptide Y (NPY) in stress and alcohol dependence. *Rev. Neurosci.* 13 85–94. 10.1515/revneuro.2002.13.1.85 12013027

[B19] Herman M. A. Cruz M. T. Sahibzada N. Verbalis J. Gillis R. A. (2009). GABA signaling in the nucleus tractus solitarius sets the level of activity in dorsal motor nucleus of the vagus cholinergic neurons in the vagovagal circuit. *Am. J. Physiol. Gastrointest. Liver Physiol.* 296 G101–G111. 10.1152/ajpgi.90504.2008 19008339 PMC2636929

[B20] Hirsch D. Zukowska Z. (2012). NPY and stress 30 years later: the peripheral view. *Cell Mol. Neurobiol.* 32 645–659. 10.1007/s10571-011-9793-z 22271177 PMC3492947

[B21] Hurtak J. J. (2002). An overview of acupuncture medicine. *J. Altern Complement Med.* 8 535–538. 10.1089/107555302320825020 12470432

[B22] Ishiguchi T. Amano T. Matsubayashi H. Tada H. Fujita M. Takahashi T. (2001). Centrally administered neuropeptide Y delays gastric emptying via Y2 receptors in rats. *Am. J. Physiol. Regul. Integr. Comp. Physiol.* 281 1522–1530. 10.1152/ajpregu.2001.281.5.R1522 11641124

[B23] Iwa M. Nakade Y. Pappas T. N. Takahashi T. (2006). Electroacupuncture elicits dual effects: stimulation of delayed gastric emptying and inhibition of accelerated colonic transit induced by restraint stress in rats. *Dig. Dis. Sci.* 51 1493–1500. 10.1007/s10620-006-9083-7 16868821

[B24] Kas M. J. Bruijnzeel A. W. Haanstra J. R. Wiegant V. M. Adan R. A. (2005). Differential regulation of agouti-related protein and neuropeptide Y in hypothalamic neurons following a stressful event. *J. Mol. Endocrinol.* 35 159–164. 10.1677/jme.1.01819 16087729

[B25] Kask A. Harro J. von Horsten S. Redrobe J. P. Dumont Y. Quirion R. (2002). The neurocircuitry and receptor subtypes mediating anxiolytic-like effects of neuropeptide Y. *Neurosci. Biobehav. Rev.* 26 259–283. 10.1016/s0149-7634(01)00066-5 12034130

[B26] Kinn Rød A. M. Harkestad N. Jellestad F. K. Murison R. (2017). Comparison of commercial ELISA assays for quantification of corticosterone in serum. *Sci. Rep.* 7:6748. 10.1038/s41598-017-06006-4 28751685 PMC5532291

[B27] Kormos V. Gaszner B. (2013). Role of neuropeptides in anxiety, stress, and depression: from animals to humans. *Neuropeptides* 47 401–419. 10.1016/j.npep.2013.10.014 24210138

[B28] Lee B. Shim I. Lee H. J. Yang Y. Hahm D. H. (2009). Effects of acupuncture on chronic corticosterone-induced depression-like behavior and expression of neuropeptide Y in the rats. *Neurosci. Lett.* 453 151–156. 10.1016/j.neulet.2009.01.076 19429024

[B29] Lenz H. J. Raedler A. Greten H. Vale W. W. Rivier J. E. (1988). Stress-induced gastrointestinal secretory and motor responses in rats are mediated by endogenous corticotropin-releasing factor. *Gastroenterology* 95 1510–1517. 10.1016/s0016-5085(88)80070-2 2846402

[B30] Levy R. L. Olden K. W. Naliboff B. D. Bradley L. A. Francisconi C. Drossman D. A. (2006). Psychosocial aspects of the functional gastrointestinal disorders. *Gastroenterology* 130 1447–1458. 10.1053/j.gastro.2005.11.057 16678558

[B31] Li Y. F. Jackson K. L. Stern J. E. Rabeler B. Patel K. P. (2006). Interaction between glutamate and GABA systems in the integration of sympathetic outflow by the paraventricular nucleus of the hypothalamus. *Am. J. Physiol. Heart Circ. Physiol.* 291 H2847–H2856. 10.1152/ajpheart.00625.2005 16877560

[B32] McDougall S. J. Widdop R. E. Lawrence A. J. (2005). Differential gene expression in WKY and SHR brain following acute and chronic air-puff stress. *Brain Res. Mol. Brain Res.* 133 329–336. 10.1016/j.molbrainres.2004.10.010 15710252

[B33] Middlekauff H. R. Hui K. Yu J. L. Hamilton M. A. Fonarow G. C. Moriguchi J. (2002). Acupuncture inhibits sympathetic activation during mental stress in advanced heart failure patients. *J. Cardiac Fail.* 8 399–406. 10.1054/jcaf.2002.129656 12528093

[B34] Molosh A. I. Sajdyk T. J. Truitt W. A. Zhu W. Oxford G. S. Shekhar A. (2013). NPY Y1 receptors differentially modulate GABAA and NMDA receptors via divergent signal-transduction pathways to reduce excitability of amygdala neurons. *Neuropsychopharmacology* 38 1352–1364. 10.1038/npp.2013.33 23358240 PMC3656378

[B35] Morales-Medina J. C. Dumont Y. Quirion R. (2010). A possible role of neuropeptide Y in depression and stress. *Brain Res.* 1314 194–205. 10.1016/j.brainres.2009.09.077 19782662

[B36] Muroya S. Funahashi H. Uramura K. Shioda S. Yada T. (2005). Gamma aminobutyric acid regulates glucosensitive neuropeptide Y neurons in arcuate nucleus via A/B receptors. *Neuroreport* 16 897–901. 10.1097/00001756-200506210-00005 15931058

[B37] Nakade Y. Tsuchida D. Fukuda H. Iwa M. Pappas T. N. Takahashi T. (2005). Restraint stress delays solid gastric emptying via a central CRF and peripheral sympathetic neuron in rats. *Am. J. Physiol. Regul. Integr. Comp. Physiol.* 288 R427–R432. 10.1152/ajpregu.00499.2004 15458973

[B38] Paxinos G. Watson C. (1997). *The Rat Brain in Stereotaxic Coordinates*, 3rd Edn San Francisco, CA: Academic, 1997.

[B39] Primeaux S. D. Wilson S. P. Cusick M. C. York D. A. Wilson M. A. (2005). Effects of altered amygdalar neuropeptide Y expression on anxiety-related behaviors. *Neuropsychopharmacology* 30 1589–1597. 10.1038/sj.npp.1300705 15770236

[B40] Pu S. Jain M. R. Horvath T. L. Diano S. Kalra P. S. Kalra S. P. (1999). Interactions between neuropeptide Y and gamma-aminobutyric acid in stimulation of feeding: a morphological and pharmacological analysis. *Endocrinology* 140 933–940. 10.1210/endo.140.2.6495 9927326

[B41] Rasmusson A. M. Schnurr P. P. Zukowska Z. Scioli E. Forman D. E. (2010). Adaptation to extreme stress: posttraumatic stress disorder, neuropeptide Y and metabolic syndrome. *Exp. Biol. Med. (Maywood)* 235 1150–1162. 10.1258/ebm.2010.009334 20881319

[B42] Reichmann F. Holzer P. (2016). Neuropeptide Y. A stressful review. *Neuropeptides* 55 99–109. 10.1016/j.npep.2015.09.008 26441327 PMC4830398

[B43] Sabban E. L. Alaluf L. G. Serova L. I. (2016). Potential of neuropeptide Y for preventing or treating post-traumatic stress disorder. *Neuropeptides* 56 19–24. 10.1016/j.npep.2015.11.00 26617395

[B44] Serova L. I. Tillinger A. Alaluf L. G. Laukova M. Keegan K. Sabban E. L. (2013). Single intranasal neuropeptide Y infusion attenuates development of PTSD-like symptoms to traumatic stress in rats. *Neuroscience* 236 298–312. 10.1016/j.neuroscience.2013.01.040 23376740

[B45] Sun J. Wu X. Meng Y. Cheng J. Ning H. Peng Y. (2015). Electro-acupuncture decreases 5-HT, CGRP and increases NPY in the brain-gut axis in two rat models of Diarrhea-predominant irritable bowel syndrome(D-IBS). *BMC Complement Altern Med.* 15:340. 10.1186/s12906-015-0863-5 26419631 PMC4589130

[B46] Sweerts B. W. Jarrott B. Lawrence A. J. (2001). The effect of acute and chronic restraint on the central expression of prepro-neuropeptide Y mRNA in normotensive and hypertensive rats. *J. Neuroendocrinol.* 13 608–617. 10.1046/j.1365-2826.2001.00674.x 11442775

[B47] Tache Y. Bonaz B. (2007). Corticotropin-releasing factor receptors and stress-related alterations of gut motor function. *J. Clin. Invest.* 117 33–40. 10.1172/JCI30085 17200704 PMC1716215

[B48] Talley N. J. Holtmann G. Walker M. M. (2015). Therapeutic strategies for functional dyspepsia and irritable bowel syndrome based on pathophysiology. *J. Gastroenterol.* 50 601–613. 10.1007/s00535-015-1076-x 25917563

[B49] Thorsell A. Carlsson K. Ekman R. Heilig M. (1999). Behavioral and endocrine adaptation, and up-regulation of NPY expression in rat amygdale following repeated restraint stress. *Neuroreport* 10 3003–3007. 10.1097/00001756-199909290-00024 10549813

[B50] Thorsell A. Svensson P. Wiklund L. Sommer W. Ekman R. Heilig M. (1998). Suppressed neuropeptide Y (NPY) mRNA in rat amygdala following restraint stress. *Regul. Pept.* 7 247–254. 10.1016/s0167-0115(98)00075-5 9802416

[B51] World Health Organization (2003). *Acupuncture: Review and Analysis Reports on Controlled Clinical Trials. WHO Library Cataloguing-in-Publication Data.* Geneva: WHO, 1–87.

[B52] Yang C. H. Lee B. B. Jung H. S. Shim I. Roh P. U. Golden G. T. (2002). Effect of electroacupuncture on response to immobilization stress. *Pharmacol. Biochem. Behav.* 72 847–855. 10.1016/s0091-3057(02)00769-4 12062574

[B53] Yang Y. Babygirija R. Zheng J. Shi B. Sun W. Zheng X. (2018). Central neuropeptide Y plays an important role in mediating the adaptation mechanism against chronic stress in male rats. *Endocrinology* 159 1525–1536. 10.1210/en.2018-00045 29425286

[B54] Yang Y. Yu H. Babygirija R. Shi B. Sun W. Zheng X. (2019). Intranasal administration of oxytocin attenuates stress responses following chronic complicated stress in rats. *J. Neurogastroenterol. Motil.* 25 611–622. 10.5056/jnm19065 31587552 PMC6786441

[B55] Yoshimoto S. Babygirija R. Dobner A. Ludwig K. Takahashi T. (2012). Anti-stress effects of transcutaneous electrical nerve stimulation (TENS) on colonic motility in rats. *Dig. Dis. Sci.* 57 1213–1221. 10.1007/s10620-012-2040-8 22258717

[B56] Zheng J. Babygirija R. Bülbül M. Cerjak D. Ludwig K. Takahashi T. (2010). Hypothalamic oxytocin mediates adaptation mechanism against chronic stress in rats. *Am. J. Physiol. Gastrointest Liver Physiol.* 299 G946–G953. 10.1152/ajpgi.00483.2009 20689056 PMC2957337

[B57] Zheng J. Dobner A. Babygirija R. Ludwig K. Takahashi T. (2009). Effects of repeated restraint stress on gastric motility in rats. *Am. J. Physiol. Regul. Integr. Comp. Physiol.* 296 R1358–R1365. 10.1152/ajpregu.90928.2008 19261914

[B58] Zhou J. Li S. Wang Y. Foreman R. D. Yin J. Zhang S. (2017). Inhibitory effects and mechanisms of electroacupuncture via chronically implanted electrodes on stress-induced gastric hypersensitivity in rats with neonatal treatment of iodoacetamide. *Neuromodulation* 20 767–773. 10.1111/ner.12602 28393479

[B59] Zukowska-Grojec Z. (1995). Neuropeptide Y. A novel sympathetic stress hormone and more. *Ann. N. Y. Acad. Sci.* 771 219–233. 10.1111/j.1749-6632.1995.tb44683.x 8597401

